# Bilateral Upper Lobe Pulmonary Oedema and Primary Mitral Regurgitation

**DOI:** 10.7759/cureus.32347

**Published:** 2022-12-09

**Authors:** Aung Hein, Yin H Wai

**Affiliations:** 1 Department of Cardiology, University Hospitals Dorset NHS Foundation Trust, Bournemouth, GBR; 2 Department of Respiratory Medicine, Poole General Hospital, Poole, GBR

**Keywords:** non-cardiogenic pulmonary oedema, upper lobes, valvular heart disease, mitral regurgitation, pulmonary oedema

## Abstract

Pulmonary oedema of uncertain aetiology is a diagnostic challenge to clinicians worldwide. Many indicators are proposed to differentiate between cardiogenic and non-cardiogenic pulmonary oedema. Mixed pulmonary oedema is an overlap between high hydrostatic pressure and increased permeability at the microvascular level. In our case, a 77-year-old patient presented with a nine-day history of shortness of breath. He was hypoxemic in the emergency department, had a pan-systolic murmur on auscultation, and blood results showed raised inflammatory markers without any fever. His chest X-ray and computed tomography pulmonary angiogram showed asymmetric pulmonary oedema in bilateral superior lobes and bilateral pleural effusions. Point-of-care echocardiography revealed severe mitral regurgitation. Trans-oesophageal echocardiography confirmed mitral valve prolapse with the chordae rupture and systolic vein reversal flow seen in the right superior pulmonary vein. He was treated with antibiotics and diuretics. After starting intravenous diuretics, there was a rapid symptomatic improvement, and a repeat chest X-ray showed significant improvements. We concluded that it was a case of mixed pulmonary oedema with predominant cardiac aetiology, and he was referred to cardiothoracic surgery for mitral valve replacement. The case showed that mixed pulmonary oedema with atypical chest radiography appearances would be a diagnostic challenge for clinicians. In such presentations, both cardiogenic and non-cariogenic causes of pulmonary oedema should be considered.

## Introduction

Acute pulmonary oedema is a medical emergency and is generally classified into cardiogenic and non-cardiogenic pulmonary oedema. In cardiogenic pulmonary oedema, increased hydrostatic pressure at the microvascular level leads to alveolar oedema. Increased alveolar capillary permeability of alveolar capillaries usually leads to non-cardiogenic pulmonary oedema [1]. In non-cardiogenic pulmonary oedema, inflammation interrupts the integrity of the alveolar-capillary membrane and is associated with the release of pro-inflammatory cytokines [2,3]. Cardiogenic causes include left ventricular failure due to coronary artery disease, valvular heart disease, congestive cardiac failure, cardiac arrhythmias, cardiomyopathies, and right to left shunts. Among cardiogenic causes related to left heart valve failure, mitral regurgitation is a well-documented cause of cardiogenic pulmonary oedema. Mitral regurgitation is a common valvular heart disease, accounting for 9.3% of the population aged over 75 years [4]. Usual symptoms include symptoms of heart failure such as shortness of breath with a variable time of onset depending on the nature and cause of mitral regurgitation, fatigue, paroxysmal nocturnal dyspnoea, orthopnoea, and dependent oedema. On chest radiography, peri-bronchial cuffing, peri-hilar haziness, septal lines, airspace opacification, and upper lobe vein diversions are common features [5]. In the literature, there are documented cases of unilateral pulmonary oedema associated with mitral regurgitation [6]. Mitral regurgitation is classified into primary and secondary mitral regurgitation. Primary mitral regurgitation is a pathology affecting the mitral valve apparatus. Causes include the degenerative process with fibroelastic deficiency or Barlow’s disease, infective endocarditis, connective tissue diseases, and rheumatic heart disease which is still often a common cause for all valve diseases in Europe, accounting for 22% [7]. Secondary mitral regurgitation is either due to changes in left atrial and ventricular chamber dilatation or ischemia. Treatment of pulmonary oedema varies according to the underlying aetiology, and it is, therefore, important to get a correct diagnosis promptly.

## Case presentation

A 77-year-old male patient presented to the emergency department with a nine-day history of gradual onset of fatigue, breathlessness, cough, and orthopnoea. He denied chest pain, palpitation, haemoptysis, cough with expectoration, and fever. Prior to this presentation, he was able to perform his activities of daily living independently. He had a history of deep vein thrombosis, and he was on edoxaban 60 mg once daily. He did not have any family history of cardiovascular disease. He had no history of smoking or alcohol consumption. He lived with his wife.

On arrival, his blood pressure was 129/79 mmHg with a heart rate of 112 beats per minute, respiratory rate of 40 breaths per minute, oxygen saturation of 96% on 4 L/minute via nasal cannula, a temperature of 36.4°C, and Glasgow Coma Scale score of 15/15. On examination, he had bilateral lung crackles at his lung bases and a grade 3 pan-systolic murmur was heard in the mitral area. His jugular venous pressure was 4 cm, without palpable liver edge, and there was no leg oedema. Physical examination of other systems was normal. Bedside arterial blood gas analysis showed that the patient was hypoxemic, and other results were unremarkable (Table 1).

**Table 1:** Arterial blood gas. Arterial blood gas on 4 L/minute (approximately FiO_2_ 36%) of oxygen. FiO_2_ = fraction of inspired oxygen

	Values	Reference range
pH	7.44	7.35–7.45
pCO_2_	4.88 kPa	4.66–6.38 kPa
pO_2_	9.92 kPa	11.1–14.4 kPa
Haemoglobin	140 g/L	120–160 g/L
sO_2_	94.6%	95.0–98.0%
Lactate	0.9 mmol/L	0.5–2.2 mmol/L
Base excess	1.0	-2.0–3.0
HCO_3_^-^	25.4 mmol/L	22.0–26.0 mmol/L

**Figure 1:**
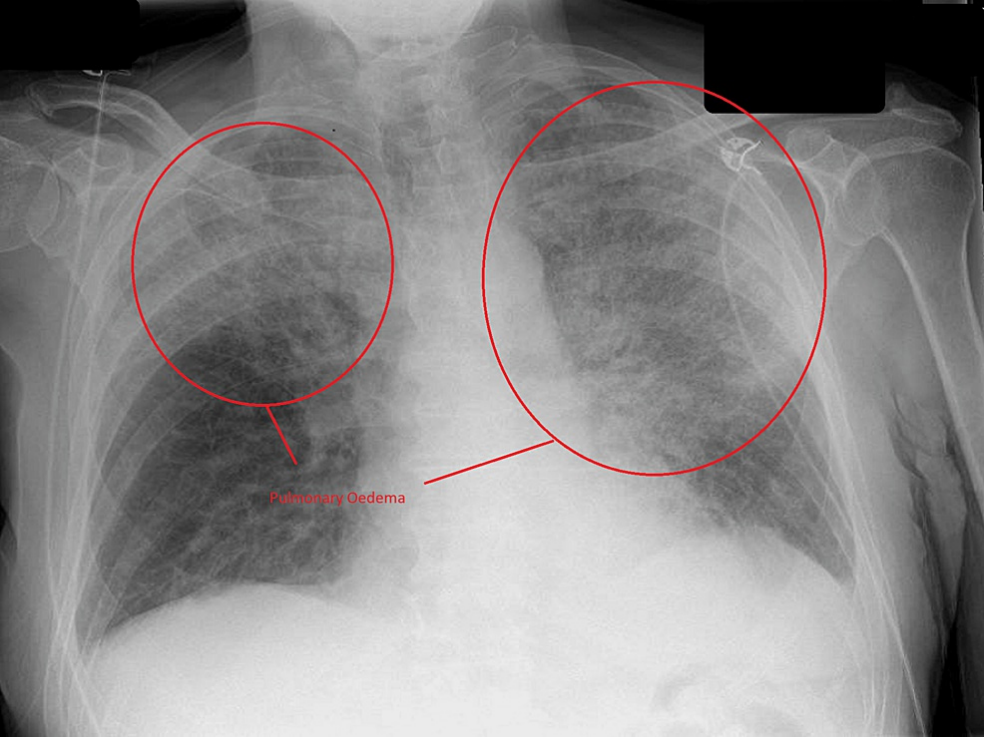
Chest X-ray. Chest X-ray with pulmonary oedema at the right upper, left upper, and middle zones.

**Figure 2:**
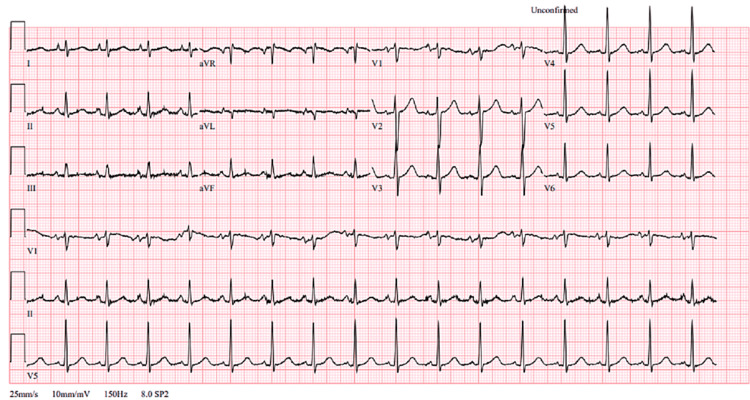
Electrocardiogram. Sinus rhythm with no ST-segment or T-wave changes.

He underwent an urgent chest X-ray which showed pulmonary oedema at the right upper zone and left upper zone extending to the middle zone (Figure 1). An electrocardiogram (ECG) showed sinus rhythm without ST-segment or T-wave changes (Figure 2).

**Figure 3:**
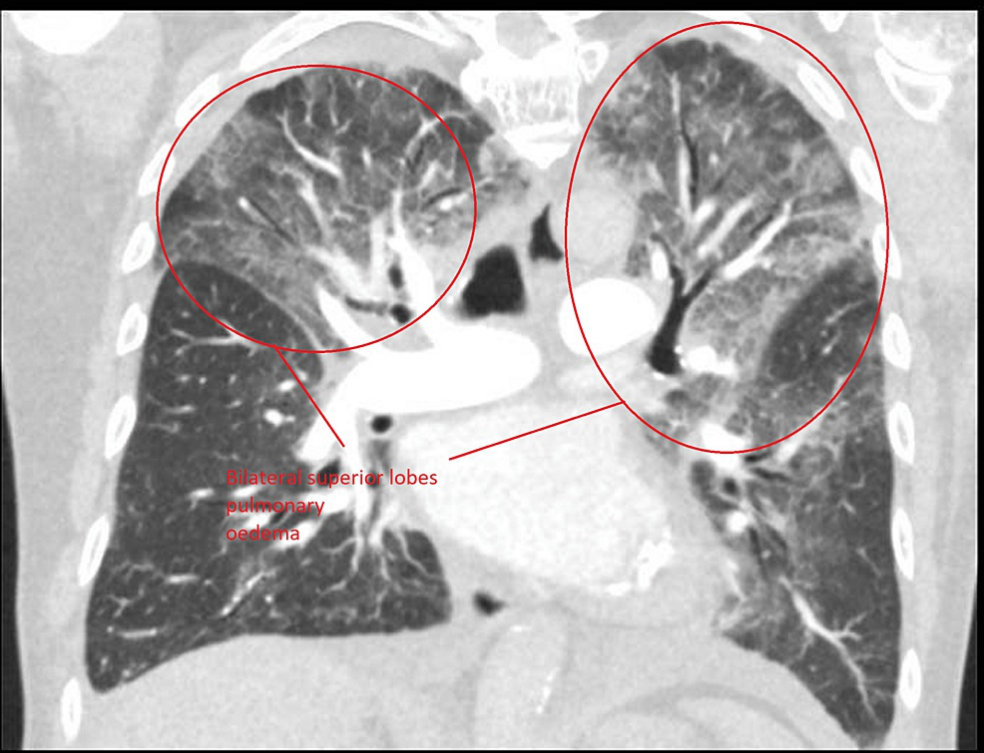
Computed tomography pulmonary angiogram. Computed tomography pulmonary angiogram did not show any pulmonary embolus but bilateral superior lobe pulmonary oedema.

Because it was in the early days of the coronavirus disease 2019 (COVID-19) pandemic, the distribution pattern of pulmonary oedema led us to think of differentials including COVID-19 pneumonitis, other atypical pneumonia, or cardiac failure. To obtain more structural information about the lungs, we performed computed tomography pulmonary angiogram (CTPA. CTPA showed that the patient had extensive ground-glass changes, with vascular dilatation and bilateral pleural effusions, which were suggestive of heart failure, and there was no evidence of pulmonary embolism. However, the radiologist advised that it could be pneumonitis or atypical infection as well. Moreover, it confirmed bilateral superior lobe pulmonary oedema (Figure 3).

**Figure 4:**
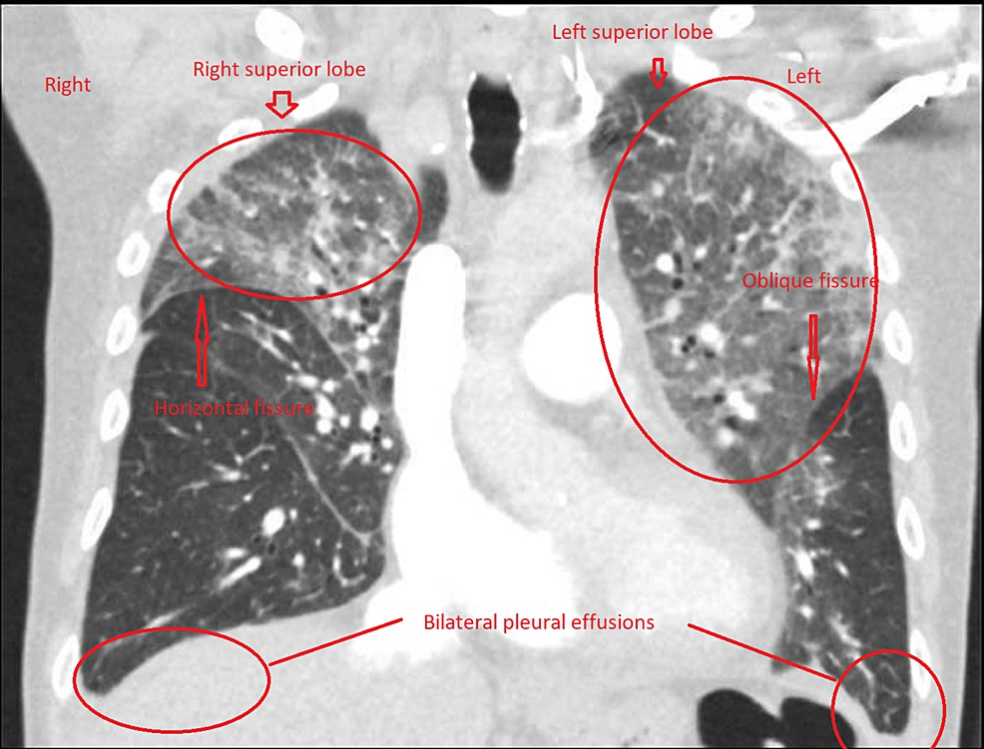
Computed tomography pulmonary angiogram coronal plane. Computed tomography pulmonary angiogram showing pulmonary oedema involving bilateral superior lobes and pleural effusions.

**Figure 5:**
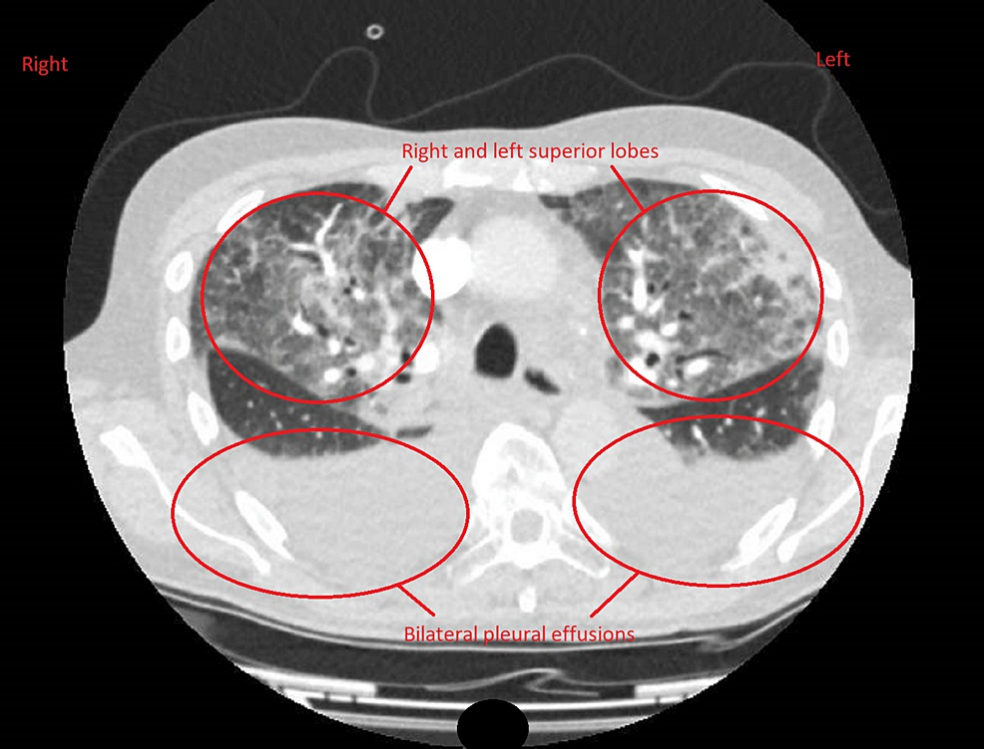
CT pulmonary angiogram axial plane CT pulmonary angiogram showing pulmonary oedema involving bilateral superior lobes and bilateral pleural effusions

Interestingly, pulmonary oedema was seen in upper zones on the CTPA lung window, and it is likely that the superior lobes of both lungs were predominantly involved (Figure 4). There were some extensions of pulmonary oedema on the left lung into the left lower lobe (Figure 5).

We sent blood for full blood count, biochemistry, liver function, coagulation, calcium, and N-terminal-pro hormone BNP (NT-proBNP). Nasal swab for COVID-19 polymerase chain reaction (PCR) was also sent. His white cell count was normal, but his C-reactive protein (CRP) was moderately high, and his BNP was also high (Table 2).

**Table 2:** Full blood count, coagulation profile, CRP, renal function, liver function, D-dimer, and serum NT-proBNP. CRP = C-reactive protein; eGFR = estimated glomerular filtration rate; MDRD = modification of diet in renal disease; NT-proBNP = N-terminal pro-B-type natriuretic peptide

Name of blood test	Day 1	Day 2	Day 3	Day 4	Day 5	Day 6	Day 7	Day 8	Reference range
Basophil count (10^9^/L)	0	0	0	0	0	0	0	0	0.0–0.1
Haematocrit (L/L)	0.4	0.39	0.39	0.39	0.37	0.36	0.37	0.4	0.40–0.50
Lymphocyte count (10^9^/L)	1.2	0.7	0.7	0.9	1	0.7	1.2	0.6	1.0–3.0
Neutrophil count (10^9^/L)	5.1	5.6	5.7	6	4.6	4	4.6	5.7	2.0–7.0
Haemoglobin estimation (g/L)	130	128	127	127	120	121	123	127	130–170
Platelet count (10^9^/L)	342	313	343	320	273	244	240	207	150–410
Mean corpuscular volume (fL)	97	98	96	95	95	94	95	98	83–101
Total white cell counts (10^9^/L)	7.5	7.4	7.1	7.9	6.5	5.6	6.9	7.2	4.0–10.0
Activated partial thromboplastin time ratio					0.95	0.92	0.88	0.9	0.85–1.15
International normalised ratio					1.36	1.31	1.23	1.2	0.90–1.10
Prothrombin time (seconds)					15	14.5	13.6	13.2	10.20–12.60
Serum CRP (mg/L)	56	47	64	58	61	95	55	63	0–9
Serum creatinine (µmol/L)	95	105	107	107	103	101	105	103	59–104
GFR calculated abbreviated MDRD (mL/minute/1.73m^2^)	67	59	58	58	61	62	59	61	-
Serum potassium (mmol/L)	4.1	3.9	4.8	3.6	3.7	4	3.9	3.4	3.5–5.0
Serum sodium (mmol/L)	132	129	131	132	130	130	130	129	132–146
Serum urea level (mmol/L)	11.1	10.8	9.4	8.7	8.7	8.6	8.6	8	2.5–6.7
Serum albumin (g/L)	42	43	44	36	32	32	31	34	35–48
Serum alkaline phosphatase (IU/L)	48	47	44	45	42	47	56	72	30–150
Serum ALT level (IU/L)	16	14	18	13	16		12	13	0–35
Serum total bilirubin level (µmol/L)	15			7					0–17
Serum calcium (mmol/L)					2.02	2.01	1.9	2.06	2.15–2.60
Corrected serum calcium (mmol/L)					2.19	2.18	2.08	2.2	2.15–2.60
Plasma D-dimer concentration (ng/mL)	454								0.0–243.0
Serum NT-proBNP (ng/L)	1,811								

**Figure 6:**
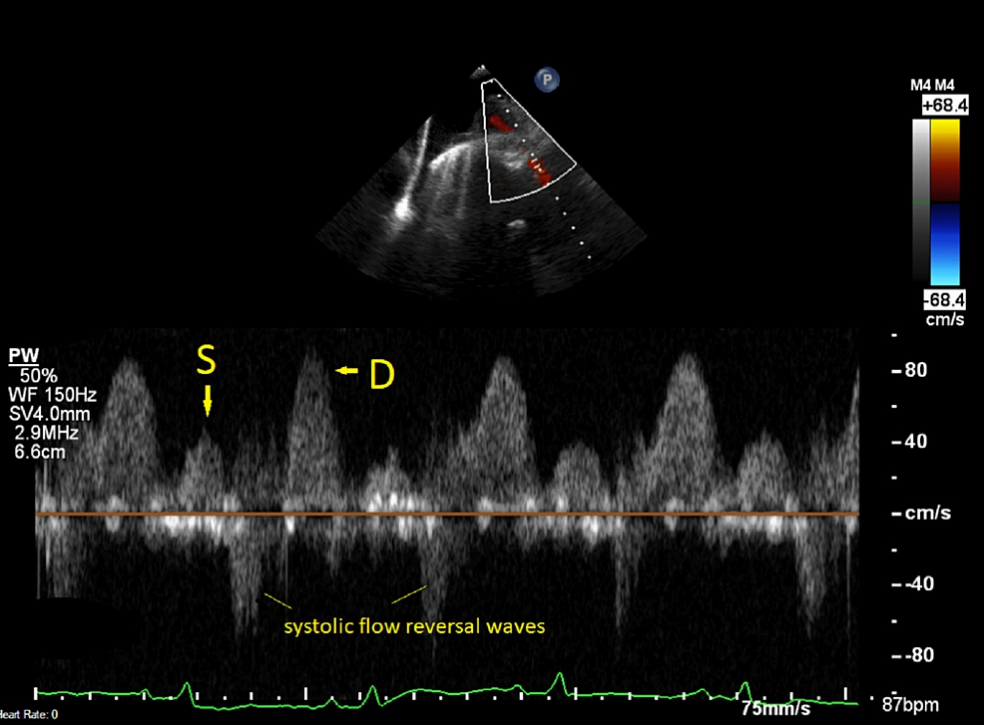
Trans-oesophageal echocardiography. Systolic flow reversal in the right superior pulmonary vein. S = systolic wave; D = diastolic wave

During the first hours in the emergency department, he was given oral dexamethasone 6 mg to cover for possible COVID-19 pneumonitis, and intravenous antibiotics were given to cover for bacterial pneumonia. Because the patient had grade 3 pan-systolic murmur and high NT-proBNP, we performed point-of-care echocardiography which revealed that the patient had severe mitral regurgitation with preserved left ventricular systolic function. After we obtained the diagnosis of mitral regurgitation, we gave him an intravenous bolus of furosemide 80 mg and discussed the case with the cardiology team. Departmental trans-thoracic echocardiography confirmed findings of point-of-care echocardiography that patient had severe mitral regurgitation, myxomatous appearance of the mitral valve, and posterior mitral valve prolapse with an ejection fraction of 72% (Video 1). Trans-oesophageal echocardiography revealed severe mitral regurgitation with prolapse of the posterior mitral valve (P2 scallop) and ruptured chordae. The left ventricle was normal in size, and a mildly impaired left ventricular ejection fraction (59%) was noted in the context of severe mitral regurgitation (Video 1). Trans-oesophageal echocardiography appearances were in keeping with fibroelastic deficiency, and systolic flow reversal was seen on the right superior pulmonary vein (Figure 6). His trans-oesophageal echocardiogram and coronary angiography were done within 72 hours of admission (Video 1).

**Video 1:** Severe mitral regurgitation. Trans-thoracic echocardiography, trans-oesophageal echocardiography, and coronary angiogram.

In the cardiac department, symptoms improved rapidly after administering intravenous diuretics. Although he never had any temperature spikes or cough, we managed to send a sputum sample, which showed normal upper respiratory tract flora only. Due to his moderately high CRP, we decided to continue antibiotics. We discussed with local microbiologists and considered virology screening. Local infection control experts did not think it would have changed the outcome of management and therefore we decided not to perform a virology screen.

**Figure 7:**
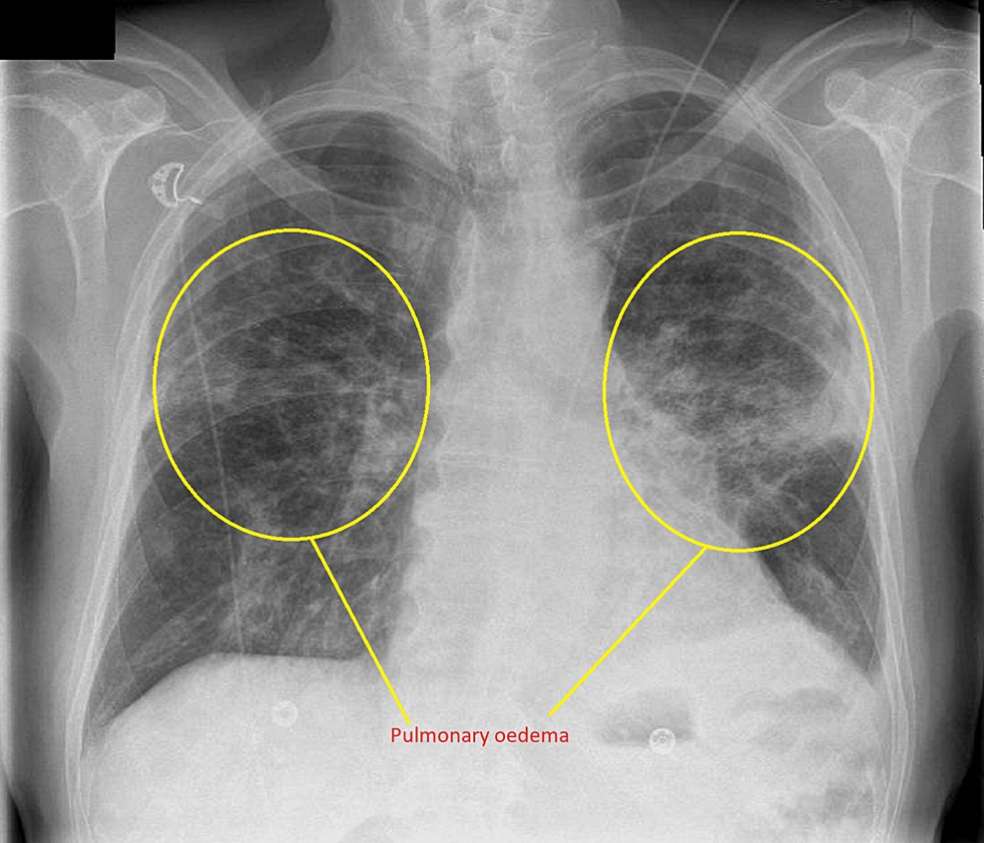
Chest X-ray. Pulmonary oedema significantly improved after the administration of intravenous diuretic.

Because the patient had severe mitral regurgitation and extensive pulmonary oedema, we administered an intravenous diuretic infusion. Repeat chest X-ray performed within 72 hours showed significant improvement (Figure 7). He was referred to cardiothoracic surgeons for mitral valve replacement.

## Discussion

Breathlessness usually accounts for 5.2% of total presentations to emergency and medical admission units in the United Kingdom [8]. Most clinicians would be familiar with common presentations of cardiopulmonary diseases such as acute coronary syndrome, pneumonia, pulmonary embolism, cardiac arrhythmia, and congestive cardiac failure. Of these pathologies, left heart valve diseases are well-documented causes of acute pulmonary oedema. The usual symptoms of such valvular heart diseases include shortness of breath, fatigue, orthopnoea, paroxysmal nocturnal dyspnoea, and leg oedema. Common examination findings are raised jugular venous pressure with the palpable liver edge, bilateral dependent oedema, pleural effusion, and bilateral crackles in both lungs. On chest X-ray, peri-hilar haziness with batwing appearances, alveolar oedema, cardiomegaly, and pleural effusions are usual findings.

In our case, the patient presented with the sub-acute onset of breathlessness without fever and cough. He had high inflammatory markers (CRP) on blood results, with bilateral crackles and grade 3 pan-systolic murmur on examination. Chest radiography features were not the typical appearance of cardiogenic pulmonary oedema. During the global pandemic, our differential diagnoses included COVID-19 pneumonitis, atypical pneumonia, or other causes of pulmonary oedema. We needed to start oral dexamethasone to cover for suspected COVID-19 pneumonitis and antibiotics for bacterial infection while awaiting the test results. CTPA provided some helpful information; however, it was unable to provide conclusive evidence to guide us on whether the patient had non-cardiogenic pulmonary oedema or cardiac failure. Point-of-care echocardiography in acute and emergency units provided very useful information to us in this scenario. After we obtained more information about the lungs from CTPA and structural abnormality from echocardiography, we decided to treat both non-cardiogenic and cardiogenic causes.

In the literature, it has been reported that mitral regurgitation of either primary or secondary causes would be associated with unilateral pulmonary oedema [6,9]. Shim et al. reported a case of bilateral upper lobe pulmonary oedema, which was thought to be related to fluid distribution [10]. After departmental trans-thoracic echocardiography and trans-oesophageal echocardiography were performed, we reviewed the images with imaging specialists. In this case, we thought that the distributing pattern of pulmonary oedema was related to regurgitant jets directed along the right and left superior pulmonary veins. On trans-oesophageal echocardiography, we identified systolic vein flow reversal in the right superior pulmonary vein, which would explain the right superior lobe pulmonary oedema. However, we were not able to obtain a doppler of the left superior pulmonary vein due to technical and patient factors. Overall, from the pattern of pulmonary oedema, we thought it was likely that the jet would have been directed in the direction of the left superior pulmonary vein.

Clinically, the patient’s response to intravenous diuretic was excellent, and he was able to mobilise in the ward with rapid symptomatic improvement within 48 hours. We reviewed whether the patient really needed antibiotics if his underlying cause was likely to be cardiogenic in origin. In the literature, there are some reports that BNP and CRP are good predictors of non-cardiogenic pulmonary oedema [11,12]. In our case, the patient had moderately high CRP without any temperature spike during his hospital stay. We sent a sputum culture, although he did not have any significant productive cough and only upper respiratory flora were reported. He underwent serial COVID-19 PCR tests, which were all negative.

In view of moderately high CRP, we cannot fully rule out a concomitant chest infection because it is not an uncommon association between pneumonia and heart failure [13]. A similar case of pulmonary oedema mimicking pneumonia on initial presentation but with mitral valvular heart disease was reported [14]. Meanwhile, we had objective evidence that the patient had primary mitral regurgitation which needed surgical intervention. Therefore, after discussion with microbiologists, we decided to continue antibiotics, notified surgeons about inflammatory markers to optimise timing for surgical intervention, and provided our opinion on the case. We also discussed with the microbiology team to consider viral screening for respiratory tract infections. We agreed that it would not have changed our management plan, and, therefore, we did not obtain a virology screen.

When the patient showed good progress, we repeated the chest X-ray, which showed obvious improvement. It provided stronger evidence that pulmonary oedema in the patient was very likely to be secondary to primary mitral regurgitation. Overall, we concluded that the case was likely mixed pulmonary oedema with a predominant cardiac problem and associated chest infection. In this case, we were first influenced by the global pandemic and our initial impressions were more inclined to infections as the most probable cause. We have learned from the case that such presentations would be related to cardiac causes as well.

## Conclusions

In clinical practice, we see multiple patients presenting with shortness of breath in emergency departments or medical admission units. It is always a challenge to clinicians when a patient presents with pulmonary oedema of unclear aetiology. In our case, the asymmetric distribution of pulmonary oedema led us to treat the patient as having pneumonia initially. Thorough history taking, physical examination, and careful analysis of signs and symptoms triggered performing useful investigations such as point-of-care echocardiography. This led to the appropriate involvement of specialists with all other necessary investigations completed in a timely fashion, with the patient receiving effective care. Despite appropriate investigations, we have learned from the case that mixed pulmonary oedema could be diagnostically challenging for clinicians, and we should consider valvular heart disease as a possible differential.
